# The Comparison of Diagnostic Ability between Blue Laser/Light Imaging and Narrowband Imaging for Sessile Serrated Lesions with or without Dysplasia

**DOI:** 10.1155/2024/2672289

**Published:** 2024-05-30

**Authors:** Reo Kobayashi, Naohisa Yoshida, Yukiko Morinaga, Hikaru Hashimoto, Yuri Tomita, Satoshi Sugino, Ken Inoue, Ryohei Hirose, Osamu Dohi, Takaaki Murakami, Yutaka Inada, Yasutaka Morimoto, Yoshito Itoh

**Affiliations:** ^1^ Department of Molecular Gastroenterology and Hepatology Kyoto Prefectural University of Medicine Graduate School of Medical Science, Kyoto, Japan; ^2^ Department of Surgical Pathology Kyoto Prefectural University of Medicine Graduate School of Medical Science, Kyoto, Japan; ^3^ Department of Gastroenterology Aiseikai Yamashina Hospital, Kyoto, Japan; ^4^ Department of Gastroenterology Kyoto First Red Cross Hospital, Kyoto, Japan; ^5^ Department of Gastroenterology Saiseikai Kyoto Hospital, Kyoto, Japan

## Abstract

**Objectives:**

Diagnostic ability of sessile serrated lesions (SSL) and SSL with dysplasia (SSLD) using blue laser/light imaging (BLI) has not been well examined. We analyzed the diagnostic accuracy of BLI for SSL and SSLD using several endoscopic findings compared to those of narrow band imaging (NBI).

**Materials and Methods:**

This was a subgroup analysis of prospective studies. 476 suspiciously serrated lesions of ≥2 mm on the proximal colon showing serrated change with magnified NBI or BLI in our institution between 2014 and 2021 were examined histopathologically. After propensity score matching, we evaluated the diagnostic ability of SSL and SSLD of the NBI and BLI groups regarding various endoscopic findings. For WLI findings, granule, depression, and reddish were examined for diagnosing SSLD. For NBI/BLI findings, expanded crypt opening (ECO) or thick and branched vessels (TBV) were examined for diagnosing SSL. Network vessels (NV) and white dendritic change (WDC) defined originally were examined for diagnosing SSLD.

**Results:**

Among matched 176 lesions, the sensitivity of lesions with either ECO or TBV for SSL in the NBI/BLI group was 97.5%/98.5% (*p* = 0.668). Those with either WDC or NV for diagnosing SSLD in the groups were 81.0%/88.9% (*p* = 0.667). Regarding the rates of endoscopic findings among 30 SSLD and 290 SSL, there were significant differences in WDC (66.4% vs. 8.6%, *p* < 0.001), NV (55.3% vs. 1.4%, *p* < 0.001), and either WDC or NV (86.8% vs. 9.0%, *p* < 0.001).

**Conclusions:**

The diagnostic ability of BLI for SSL and SSLD was not different from NBI. NV and WDC were useful for diagnosing SSLD.

## 1. Introduction

Serrated lesions of the colorectum are divided into hyperplastic polyps (HP), sessile serrated lesions (SSL), SSL with dysplasia (SSLD), and traditional serrated adenomas (TSA), according to the 2019 World Health Organization (WHO) classification [1]. SSL and SSLD lead to microsatellite instability- (MSI-) high colorectal cancer via a serrated pathway characterized by BRAF mutation and CpG island methylation phenotype, and serrated lesions except HP are considered precursors of colorectal cancer [2–6].

SSL reportedly makes up 8% of all polyps and SSL and SSLDs are indications for endoscopic treatments according to several guidelines [7–9]. SSLDs were observed in serrated lesions of <10 mm; therefore, accurate endoscopic diagnosis is necessary even for small serrated lesions [9, 10]. The endoscopic findings of SSL with white light imaging (WLI) are characterized by a fading, slightly elevated lesion with mucus on a predominantly proximal colon [9]. Type II-open pit, which is thought to indicate dilation of the crypt, is characterized by magnifying chromoendoscopy [11]. In addition, expanded crypt opening (ECO) and thick and branched vessels (TBV) are reported endoscopic findings of SSL with narrow band imaging (NBI) magnification [9, 12–16]. However, there have been no reports on the diagnostic ability for SSL of blue laser/light imaging (BLI), which is a narrow band light observation from a laser or light-emitting diode (LED) light source [17–19]. Reddish, depressions, and granular elevations are seen in SSLD on WLI, but there have been a few reports on the endoscopic findings of SSLD only on NBI magnification [20]. In this study, we analyzed the diagnostic ability of BLI for SSL and SSLD compared to that of NBI regarding each endoscopic finding. Additionally, we originally defined network vessels (NV) and white dendritic change (WDC) of NBI and BLI magnification for improving diagnostic accuracy of SSLD and evaluated their diagnostic ability.

## 2. Methods

This was a single-center, retrospective study and was a subgroup analysis of several prospective studies. Study subjects were suspiciously serrated lesions (HP, SSL, and SSLD) of ≥2 mm from the cecum to the descending colon, which showed serrated change with either magnified BLI or NBI by two expert endoscopists between June 2014 and April 2021. In this period, not to miss any SSLs and SSLDs even in small lesions < 5 mm, we performed histopathological assessment to all lesions ≥ 2 mm which have a serrated change with possibility of SSLs and SSLDs as a prospective observational study. Serrated change was endoscopic findings of none vessels or isolate lacy vessels or dark or white spots in type 1 of the Japan NBI Expert Team (JNET) classification [21]. Histopathological specimens were obtained by biopsy, cold snare polypectomy (CSP), endoscopic mucosal resection (EMR), precutting EMR, or endoscopic submucosal dissection (ESD). The inclusion criteria for colonoscopy were as follows: patients with various symptoms, cancer screening, positive fecal immunohistochemical test, and surveillance after surgical/endoscopic resection of polyps and cancers.

**Figure 1:**
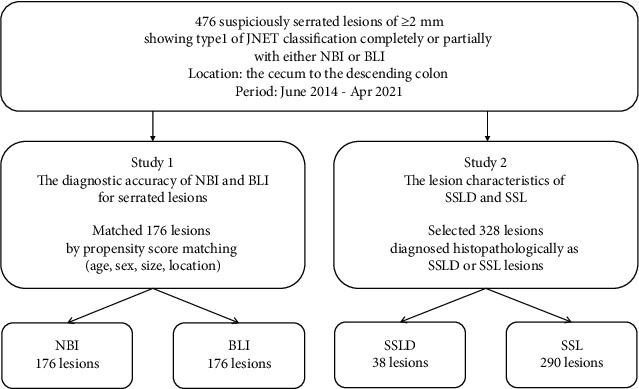
Study flow.

Two studies were performed to examine SSL and SSLD (Figure 1). In study 1, we analyzed the diagnostic ability of BLI for diagnosing SSL and SSLD compared to that of NBI. The overall 476 suspiciously serrated lesions were divided into the NBI (*n* = 310) and BLI (*n* = 196) groups (including 30 overlapping lesions observed in both NBI and BLI), and propensity score matching was performed according to age, sex, and lesion size/location. Finally, we could analyze 176 lesions in each group and calculated the sensitivity, specificity, and accuracy of various BLI/NBI endoscopic findings for diagnosing SSL and SSLD. We also examined 30 lesions observed with both NBI and BLI. The consistent rates of each endoscopic finding between NBI and BLI for diagnosing SSL and SSLD were calculated. In study 2, we analyzed 328 SSLD or SSL lesions and compared the lesion characteristics of SSLD to that of SSL. Lesion size, morphology, and various endoscopic findings of WLI and BLI/NBI were examined. In addition, we analyzed the histopathological findings using immunohistochemical examinations of adipophilin for endoscopic findings of NV and WDC in 20 SSLD lesions, in which endoscopic findings and histopathological findings were evaluated accurately.

**Figure 2:**
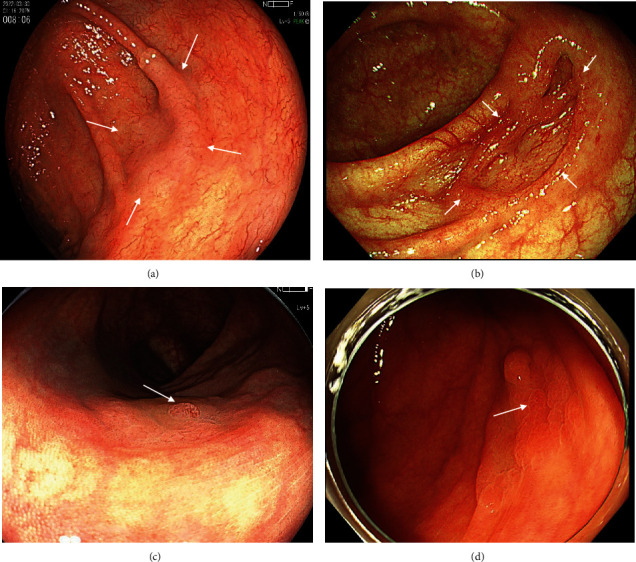
Endoscopic finding of SSL and SSLD with WLI. (a) WLI finding of SSL. A fading and slightly elevated lesion of 20 mm (white arrows). (b) WLI finding of SSLD. A lesion of 15 mm with central depression (white arrows). (c) WLI finding of SSLD. A lesion of 18 mm with a granular elevation (white arrow). (d) WLI finding of SSLD. A lesion of 18 mm with partial redness (white arrow).

**Figure 3:**
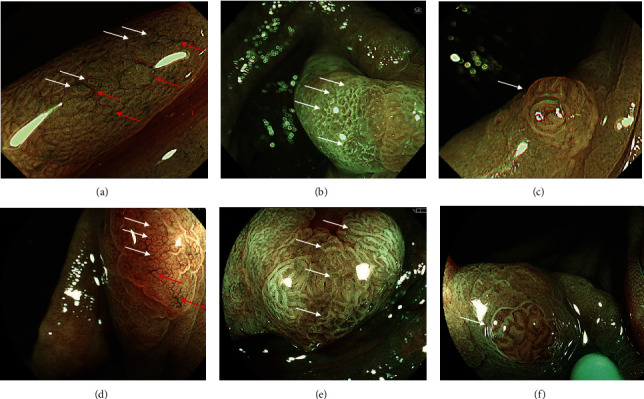
Endoscopic findings of SSL and SSLD with NBI and BLI. (a) NBI magnification finding of SSL. Expanded crypt opening (ECO) (white arrows) and thick and branched vessels (TBV) (red arrows) are observed. (b) NBI magnification finding of SSL with dysplasia (SSLD). White dendritic changes (WDC) are white dendritic and serrated network or winding-line-like structures around crypts (white arrows). (c) NBI magnification finding of SSLD. Network vessels (NV) are meshed or spiral vessels with or without irregularity around crypts (white arrow). (d) BLI magnification finding of SSL. ECO (white arrows) and TBV (red arrows) are observed. (e) BLI magnification finding of SSLD. WDC is observed (white arrows). (f) BLI magnification finding of SSLD. NV is observed (white arrow).

Regarding endoscopic findings, three endoscopic findings of WLI such as granular elevation, depression, and redness were also examined for the diagnosis of SSLD as previously reported (Figure 2) [22]. The endoscopic findings of ECO and TBV on NBI and BLI magnification were used as specific findings of SSL according to previous reports (Figure 3) [16, 19]. ECO was defined as a dot-shaped surface pattern that was dilated compared with the normal crypt. TBV was defined as a dilated vessel that did not surround crypts. Regarding endoscopic findings of SSLD on BLI and NBI magnification, two original findings, namely, NV and WDC, were analyzed. NV were defined as a definite area of ≥2 mm showing meshed, spiral, or irregular vessels around crypts corresponding to types 2A and 2B of JNET classification. WDC was defined as the presence of severe dense white structures including white dendritic and serrated network or winding-line-like structures around crypts on the superficial layer of the lesion (Figure 3) [21]. All endoscopic representative still images were reviewed by three expert endoscopists, and the majority opinion was adopted as a final diagnosis.

All procedures were performed with laser and LED endoscope systems (Fujifilm Co., Tokyo, Japan) or an endoscope system with Xenon light source (EVIS Lucera Elite, Olympus Co., Tokyo, Japan), and the scopes were EC-760ZP-V, EC-L600ZP, EC-600ZP7, and EC-660ZP for Fujifilm and CF-HQ290I and PCF-H290AZI for Olympus. We also used caps (D-201-14304, D-201-13404, MAJ-1990, Olympus Co., Tokyo, Japan) for almost all procedures. In the BLI observations, both of the laser and LED endoscopic systems were used. Our previous study showed the noninferiority of the diagnostic accuracy of BLI magnification between the laser and LED groups for colorectal lesions including SSL and SSLD [23].

Morphology of the lesions was classified into nonpolypoid and polypoid lesions according to the Paris classification [24]. All resected specimens were fixed with formalin after resection. To evaluate the margin precisely, histopathological technicians fixed the specimens to facilitate a thorough evaluation of the vertical margin while adjusting the direction of the specimens. The specimens were stained with hematoxylin and eosin and adipophilin and evaluated by an authorized pathologist (Y.M.). Histopathological diagnosis was made according to the 2010 or 2019 WHO classification [1, 25]. This research was approved by the Ethics Committee of Kyoto Prefectural University of Medicine (ERB-C-1600) and was conducted in accordance with the World Medical Association Declaration of Helsinki.

### 2.1. Statistical Analyses

The results were analyzed using the chi-squared test, Yates continuity correction, and Mann–Whitney *U* test. All statistical analyses were performed using SPSS software (version 22.0; IBM Japan, Ltd., Tokyo, Japan). Continuous variables, such as tumor size, were analyzed using the Mann–Whitney *U* test. Propensity score matching was performed to remove confounding factors. Categorical variables were analyzed using the chi-squared test. Statistical significance was set at *p* < 0.05.

## 3. Results

The 476 lesions diagnosed endoscopically as serrated lesions in 287 patients were analyzed (Table 1). The mean age (mean ± standard deviation: SD) was 65.1 ± 12.3 years, and the mean tumor size (mean ± SD) was 10.1 ± 8.1 mm. Regarding the way of observation, 280 and 166 lesions were observed with NBI and BLI, respectively, while 30 lesions were observed with both NBI and BLI. Histopathological diagnoses were 290 SSLs, 36 SSLDs, 2 T1 with SSL, 101 HPs, 17 adenomas, and 30 colitis and normal mucosa.

After propensity score matching, 176 lesions in the NBI and BLI groups were finally examined (Table 2). Lesion sizes of each group were 11.5 ± 9.8 and 13.2 ± 10.0 (*p* = 0.445). There were no significant differences in age, sex, lesion location, morphology, and histopathology.

**Figure 4:**
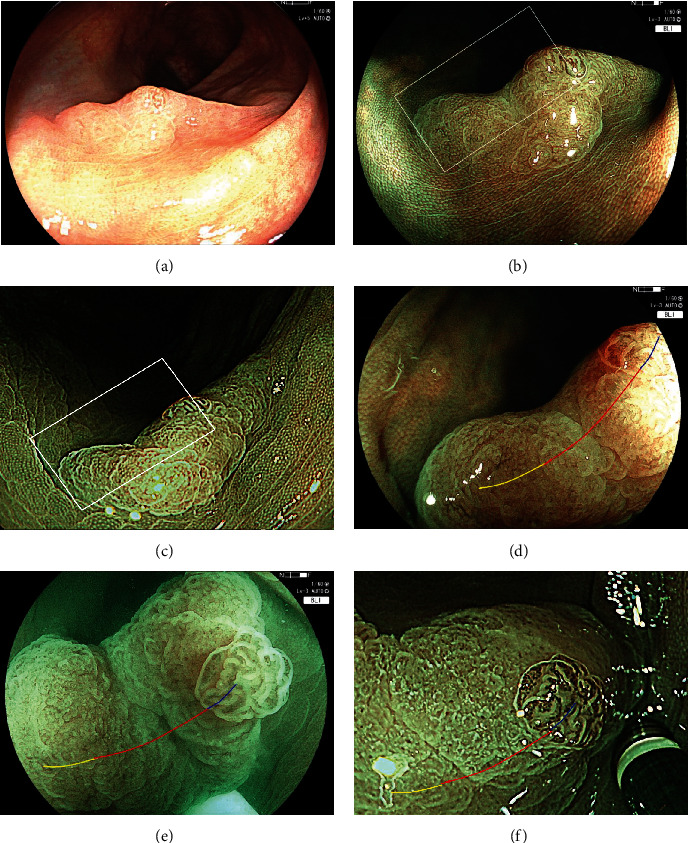
A case presentation of SSLD with WDC. (a) A SSLD of 15 mm on the descending colon with WLI. It is a fading and slightly depressed lesion with a granular elevation. (b) Image with BLI. (c) Image with NBI. (d) BLI magnification of the white box in (b). NV is observed on the granule (blue line). WDC is observed on the depressed area (red line). WDC is not observed (yellow line). (e) BLI magnification of white box under water. WDC appears whiter and clearer than the regular view under air. (f) NBI magnification of the white box in (c). NV is observed on the granule (blue line). White dendritic change is observed on the depressed area (red line), and WDC is not observed (yellow line).

The sensitivity of TBV for diagnosing SSL with BLI and NBI among matched 176 lesions was 88.8% and 85.7% (*p* = 0.460) (Table 3). The sensitivity of ECO was 98.5% vs. 95.8% (*p* = 0.259), and the sensitivity of lesions with either TBV or ECO was 98.5% vs. 97.5% (*p* = 0.668). The accuracy of lesions with either TBV or ECO of BLI was significantly higher than that of NBI (81.3% vs. 72.2%, *p* = 0.044). In the diagnosis of SSLD, the sensitivity of WDC, NV, and WDC or NV with BLI and NBI was not significantly different (WDC: 72.2% vs. 71.4%, *p* = 0.956; NV: 55.6% vs. 42.9%, *p* = 0.429; and WDC or NV: 88.9% vs. 81.0%, *p* = 0.667) (Table 3). The accuracy of lesions with either WDC or NV was also not significantly different between the two groups (93.2% vs. 85.8%, *p* = 0.055). However, specificity of WDC and WDC or NV was significantly lower in BLI than those in NBI (WDC: 86.2% vs. 95.9%, *p* = 0.018, WDC or NV: 85.3% vs. 95.9%, *p* = 0.011). We could examine 20 SSLs and 10 SSLDs observed with both NBI and BLI. The consistence rates of each endoscopic finding between BLI and NBI were 100% (30/30) for TBV, 100% (30/30) for ECO, 96.7% for WDC (29/30), and 93.3% (28/30) for NV. The diagnostic accuracy for the 30 cases between BLI and NBI was 100%/100% for SSL (*p* = 1.0) and 80.0%/83.3% for SSLD (*p* = 1.0) (Figure 4).

The comparison of the lesion characteristics of the SSLD/T1 with SSL and the SSL groups showed that there was a significant difference in tumor size (mm, mean ± SD) between the two groups (17.8 ± 10.5 vs. 11.4 ± 8.7, *p* < 0.001) (Table 4). Regarding WLI endoscopic findings, granular elevation, depression, and redness in the SSLD/T1 with SSL and the SSL groups were observed in 26.3% vs. 1.7% (*p* < 0.001), 18.4% vs. 2.1% (*p* < 0.001), and 23.7% vs. 0.7% (*p* < 0.001), respectively. Either one of the three findings was observed in 47.4% and 4.1% in the two groups (*p* < 0.001). There was no significant difference in the frequency of the use of ECO and TBV as the endoscopic findings of SSL in the two groups. Regarding the endoscopic findings of SSLD in the two groups, WDC was found in 66.4% and 8.6% (*p* < 0.001) and NV was found in 55.3% and 1.4% (*p* < 0.001). Either WDC or NV was found in 86.8% and 9.0%, respectively (*p* < 0.001).

**Figure 5:**
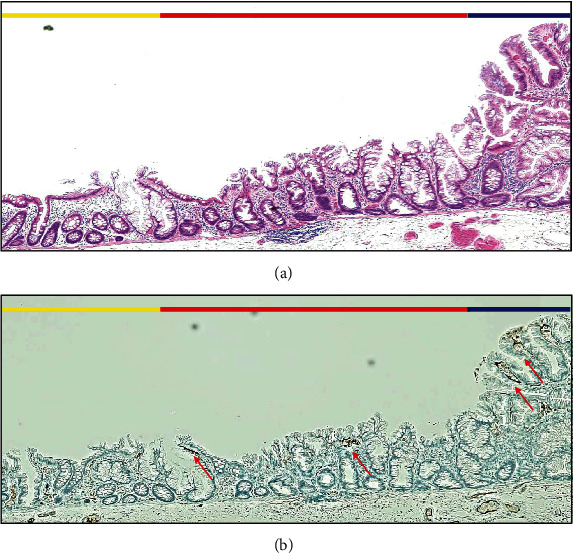
Histopathological findings of WDC in H.E. stains and immunohistochemical examination. (a) Histopathological examination with H.E. stains of blue, red, and yellow lines in Figure 4(c). The blue line corresponds to the area of dysplasia, and it does not show WDC. The red line corresponds to WDC. The yellow line corresponds to an area without WDC. In the area of WDC (red line), serrated-shaped superficial epithelium of the colonic mucosa is observed. (b) Immunohistochemical examination with adipophilin of (a) shows several minor positive areas (red arrows). In WDC area (red line), positive of adipophilin is detected. But it is also detected in the area of dysplasia (blue line).

Regarding the histopathological findings of NV and WDC, NV was consistent with dysplastic area in 18 out of 20 SSLD lesions with NV (90.0%) (Figures 4 and 5). WDC was not consistent with the dysplastic area, but it was consistent with high serrated-shaped change of superficial epithelium of the colonic mucosa and large glands with severe serration around dysplastic area in 17 out of 20 SSLD lesions with WDC (85.0%). The immunohistochemical stain of adipophilin showed that a positive area was not consistent with the area with WDC (Figure 5).

## 4. Discussion

Regarding the findings of TBV and ECO, a previous study analyzed 110 colorectal lesions of the whole colorectum showing type 1 of NICE classification with NBI [16]. The sensitivity/accuracy of diagnosing SSL about ECO was 84.3%/82.4%, and those about TBV were 45.1%/59.2%, respectively. The sensitivity of lesions when either ECO or TBV was reported to be 97.0%. BLI has also been reported to be useful in the diagnosis of serrated lesions and is noninferior to NBI [26, 27]. In the current study, the sensitivity/accuracy of ECO and TBV with NBI was 95.8%/75.0% and 85.7%/68.8%, respectively. With BLI magnification, the sensitivity/accuracy of ECO and TBV was 98.5%/82.4% and 88.8%/76.1%, respectively. Our data showed slightly better results compared to the previous study because we only analyzed lesions from the cecum to the descending colon. Additionally, the accuracy of BLI for diagnosing SSL was significantly higher than that of NBI. We suggested that it was due to the more enhancement of surface pattern and vessel pattern in BLI than those of NBI according to a previous study [17–19].

SSLD is reported to be found in 5-13% of SSL, and SSL with intramucosal carcinoma in 2%, and with T1 carcinoma in 1% [28–30]. In the current study, the ratios of SSLD and T1 carcinomas were similar to previous reports. Sano et al. analyzed 326 SSLs and SSLDs, and the mean sizes of SSL and SSLD were 9.1 and 14.3 mm, respectively. The ratios of SSLDs ≤ 5 mm, 6-9 mm, and ≥10 mm were 0%, 6.0%, and 13.6%, respectively [31]. In the current study, the mean size of SSLD was larger than that of SSL, and SSLD became more common as tumor diameter increased. The rates of SSLD were slightly higher than those in the previous study. We suggested that the reason was probably due to the location of lesions from the cecum to the descending colon in the current study. Additionally, SSLD of <5 mm was observed not in the previous study but was observed in our study, suggesting that diagnosis is important even in small lesions.

The endoscopic findings of SSLD are reported to be granular elevation, redness, and depression on WLI and neoplastic pits on chromoendoscopy [15, 21, 22, 32, 33]. Regarding WLI, Murakami et al. reviewed 462 serrated lesions and reported that the presence of at least one of the findings of pedunculated/semipedunculated, reddish, double elevation, and depression had a high sensitivity and specificity (91.7% and 85.3%) for diagnosing SSLD [22]. In the current study, the presence of at least one finding of granular elevation, depressions, and redness in WLI was only 47.4%. Regarding IEE, Tate et al. reported that NBI showed hypervascularity in 86.1% of SSLD (31/36) [32]. Murakami et al. reported the sensitivity/specificity/accuracy of types 2A, 2B, and 3 of JNET classification for SSLD and T1 cancer was 83.9%/95.5%/95.5% [33]. In the current study, the sensitivity of NV alone for SSLD was lower than previous studies using JNET classification (NBI: 42.9%, BLI: 55.6%). It was due to the definition of NV. We defined it as a definite area of ≥2 mm for preventing overdiagnosis. However, additional use of WDC could improve the sensitivity. According to the diagnosis of SSLD, our study suggested that WLI alone was not sufficient and the concurrent use of magnification of NBI or BLI was desirable. Additionally, NBI was slightly superior to BLI in SSLD diagnosis regarding specificity of WDC or NV. We suggested that this was because BLI enhanced the surface structure more than NBI and this overestimated part of WDC [17–19, 34]. According to our results, both NBI and BLI are useful in the diagnosis and treatment of SSL and SSLD, with a slightly higher accuracy for BLI in the diagnosis of SSL and a slightly higher accuracy rate for NBI in the diagnosis of SSLD, but there is no significant difference between them. However, the resolution and brightness of endoscopy increase continuously with advances in each modality and equipment, and further studies are needed in the future. This is the first report to compare the diagnostic ability for SSL/SSLD between NBI and BLI.

Regarding WDC and NV of histopathological findings, NV was consistent with dysplastic and cancerous area. On the other hand, WDC was observed in serrated high serrated-shaped change of superficial epithelium of the colonic mucosa and large glands with severe serration around dysplastic area, and WDC was not consistent with dysplastic area and T1 cancer area. However, lesions with WDC showed high sensitivities both for NBI (71.4%) and BLI (72.2%), and WDC was seen in 66.4% out of 38 SSLDs. Thus, we hypothesized that WDC was indirect findings for diagnosing SSLD. Additionally, white opaque substance (WOS) is a well-known whitish change of colonic mucosa detected in adenoma, SSL, and HP, and it was positive for the immunohistochemical stain of adipophilin [35, 36]. WDC was considered to be related with WOS because stain of adipophilin was also seen in WDC area. However, WDC area and positive of adipophilin were not consistent perfectly. Further analysis about the analysis of WDC and the comparison between WDC and WOS should be performed.

The limitation of this study was that it was a single-center retrospective study. The lesions included in the study were serrated lesions located in the cecum to descending colon. All lesions were diagnosed endoscopically only by expert endoscopists. Serrated lesions without type 1 of JNET classification were not included. A preprint has previously been published in Research Square automatically [37].

## 5. Conclusions

In this study, we found that the diagnostic ability of BLI was as high as that of NBI for diagnosing SSL and SSLD though there were slight differences between BLI and NBI. In addition, WDC and NV were useful for the diagnosing SSLD.

## Figures and Tables

**Table 1:** Lesion characteristics of all lesions.

Lesion number	476
Patient number	287
Age, mean ± SD (range)	65.1 ± 12.3 (25-85)
Sex, % (*n*) male/female	44.3/55, 7 (127/160)
Lesion size (mm), mean ± SD (range)	10.1 ± 8.1 (2-50)
Lesion size, % (*n*) (≤5, 6-10, 11-20, ≥21)	39.9/29.4/21.6/9.0 (190/140/103/43)
Method of observation (NBI/BLI/NBI and BLI)	58.8/34.9/6.3 (280/166/30)
Method of BLI observation (laser/LED/laser and LED)	70.4/23.0/6.6 (138/45/13)
Location, % (*n*) C/A/T/D	23.5/34.5/33.4/8.6 (112/164/159/41)
Morphology, % (*n*) polypoid/non-polypoid	14.1/85.9 (67/409)
Histopathology, % (*n*) SSL/SSLD/T1 with SSL/HP/adenoma/others	60.9/7.6/0.4/21.2/3.6/6.3 (290/36/2/101/17/30)
Histopathology of SSL, % (*n*) SSL/SSLD/T1 with SSL	88.4/11.0/0.6 (290/36/2)

SD: standard deviation; NBI: narrowband imaging; BLI: blue laser/light imaging; LED: light-emitting diode; C: cecum; A: ascending colon; T: transverse colon; D: descending colon; SSL: sessile serrated lesions; SSLD: SSL with dysplasia; HP: hyperplastic polyp.

**Table 2:** The characteristics of serrated lesions on NBI and BLI before and after propensity score matching.

	Before matching		ASD	*p* value	After matching		ASD	*p* value
	NBI (*n* = 310)	BLI (*n* = 196)			NBI (*n* = 176)	BLI (*n* = 176)		
Age, mean ± SD (range)	65.3 ± 12.2 (25-85)	65.1 ± 12.5 (29-85)	0.015	0.907	64.7 ± 11.56 (34-85)	64.4 ± 12.3 (29-85)	0.015	0.442
Sex, male/female, % (*n*)	44.1/55.9 (90/114)	41.3/58.7 (43/61)	0.182	0.904	43.8/56.3 (77/99)	39.8/60.2 (70/106)	0.066	0.449
Lesion location, % (*n*) C/A/T/D	23.9/34.8/31.0/10.3 (74/108/96/32)	25.5/33.2/36.2/5.1 (50/65/71/10)	0.060	0.159	25.0/29.0/34.7/11.4 (44/51/61/20)	24.4/33.0/36.9/5.7 (43/58/65/10)	0.076	0.270
Morphology, % (*n*) Polypoid/nonpolypoid	12.6/87.4 (39/271)	9.7/90.3 (19/177)		0.320	13.1/86.9 (23/153)	10.8/89.2 (19/157)		0.511
Lesion size (mm), mean ± SD (range)	9.0 ± 8.1 (2-50)	14.4 ± 10.4 (2-50)	0.497	<0.001	11.5 ± 9.8 (2-50)	13.2 ± 10.0 (2-50)	0.139	0.445
Lesion size, % (*n*) (≤5, 6-10, 11-20, ≥21)	44.2/33.5/15.1/7.1 (137/104/47/22)	27.0/18.4/34.7/19.9 (53/36/68/39)	—	—	33.5/29.5/24.4/12.5 (59/52/43/22)	30.1/20.5/33.5/15.9 (53/36/59/28)	—	—
Histopathology, % (*n*) SSL/SSLD (+T1)/HP/adenoma/others	57.4/8.4/22.3/5.2/6.8 (178/26/69/16/21)	67.3/11.2/16.3/0.5/4.6 (132/22/32/1/9)		0.672	55.7/11.9/20.5/6.8/5.1 (98/21/36/12/9)	65.9/10.2/18.2/0.6/5.1 (116/18/32/1/9)		0.354

SD: standard deviation; C: cecum; A: ascending colon; T: transverse colon; D: descending colon; SSL: sessile serrated lesions; SSLD: SSL with dysplasia; HP: hyperplastic polyp; ASD: absolute standardized difference.

**Table 3:** Comparison of the diagnostic capability of NBI and BLI for SSL and SSLD.

SSL		NBI	BLI	*p* value
TBV	Sensitivity, % [95% CI] (*n*)	94.1 [88.1-97.3] (112/119)	88.8 [82.2-93.2] (119/134)	0.134
	Specificity, % [95% CI] (*n*)	33.3 [22.4-46.3] (19/57)	35.7 [22.9-50.8] (15/42)	0.805
	Accuracy, % [95% CI] (*n*)	68.8 [61.5-75.1] (121/176)	76.1 [69.2-81.8] (134/176)	0.121

ECO	Sensitivity, % [95% CI] (*n*)	95.8 [90.2-98.4] (114/119)	98.5 [94.3-99.9] (132/134)	0.259
	Specificity, % [95% CI] (*n*)	31.6 [20.9-44.5] (18/57)	31.0 [18.9-46.1] (13/42)	0.947
	Accuracy, % [95% CI] (*n*)	75.0 [68.0-80.8] (132/176)	82.4 [76.0-87.3] (145/176)	0.090

TBV or ECO	Sensitivity, % [95% CI] (*n*)	97.5 [92.5-99.4] (116/119)	98.5 [94.3-99.9] (132/134)	0.668
	Specificity, % [95% CI] (*n*)	19.3 [10.9-31.5] (11/57)	26.2 [15.1-41.2] (11/42)	0.415
	Accuracy, % [95% CI] (*n*)	72.2 [65.1-78.2] (127/176)	81.3 [74.8-86.3] (143/176)	0.044

SSLD		NBI	BLI	*p* value
WDC	Sensitivity, % [95% CI] (*n*)	71.4 [49.7-86.4] (15/21)	72.2 [48.8-87.8] (13/18)	0.956
	Specificity, % [95% CI] (*n*)	95.9 [89.6-98.7] (94/98)	86.2 [78.6-91.4] (100/116)	0.018
	Accuracy, % [95% CI] (*n*)	91.5 [85.0-95.5] (109/119)	84.3 [77.1-89.5] (113/134)	0.078

NV	Sensitivity, % [95% CI] (*n*)	42.9 [24.4-63.4] (9/21)	55.6 [33.6-75.4] (10/18)	0.429
	Specificity, % [95% CI] (*n*)	100 [95.5-100.0] (98/98)	96.6 [91.1-98.9] (112/116)	0.127
	Accuracy, % [95% CI] (*n*)	89.9 [83.0-94.2] (107/119)	91.0 [84.8-94.9] (122/134)	0.760

WDC or NV	Sensitivity, % [95% CI] (*n*)	81.0 [59.4-92.9] (17/21)	88.9 [65.9-98.1] (16/18)	0.667
	Specificity, % [95% CI] (*n*)	95.9 [89.6-98.7] (94/98)	85.3 [77.6-90.7] (99/116)	0.011
	Accuracy, % [95% CI] (*n*)	93.2 [87.1-96.7] (111/119)	85.8 [78.8-90.8] (115/134)	0.055

SSL: sessile serrated lesions; SSLD: SSL with dysplasia; TBV: thick and branched vessels; ECO: expanded crypt opening; WDC: white dendritic change; NV: network vessels.

**Table 4:** Comparison of lesion characteristics between SSL and SSLD.

	SSLD T1 with SSL	SSL	*p* value
Lesion number	38	290	
Patient number	20	181	
Age, mean ± SD (range)	67.2 ± 14.8 (28-85)	65.0 ± 12.6 (25-83)	0.533
Sex, % (*n*) male/female	50/50 (10/10)	39.8/60.2 (72/109)	0.576
Lesion size (mm), mean ± SD (range)	17.8 ± 10.5 (3-50)	11.4 ± 8.7 (2-50)	<0.001
Lesion size, % (*n*) (≤5, 6-10, 11-20, ≥21)	10.5/23.7/34.2/31.6 (4/9/13/12)	31.7/29.7/27.9/10.7 (92/86/81/31)	
Location, % (*n*) C/A/T/D	28.9/39.5/26.3/5.3 (11/15/10/2)	26.2/35.5/31.0/7.2 (76/103/90/21)	0.879
Morphology, % (*n*) polypoid/non-polypoid	10.5/89.5 (4/34)	10.3/89.7 (30/260)	0.580
Method of observation (NBI/BLI/NBI and BLI)	16/12/10	158/112/20	

WLI endoscopic findings			
Granular elevation	26.3 (10)	1.7 (5)	<0.001
Depression	18.4 (7)	2.1 (6)	<0.001
Redness	23.7 (9)	0.7 (2)	<0.001
Either of 3 findings	47.4 (12)	4.1 (12)	<0.001

NBI/BLI endoscopic findings			
ECO (overall)	100 (38)	96.2 (279)	0.252
TBV (overall)	81.6 (31)	84.8 (246)	0.634
Either of ECO or TBV	100.0 (38)	98.6 (286)	0.951
WDC (overall)	66.4 (26)	8.6 (25)	<0.001
NV (overall)	55.3 (21)	1.4 (4)	<0.001
Either of WDC or NV	86.8 (33)	9.0 (26)	<0.001

SD: standard deviation; SSL: sessile serrated lesions; SSLD: SSL with dysplasia; NBI: narrowband imaging; BLI: blue laser/light imaging; C: cecum; A: ascending colon; T: transverse colon; D: descending colon; HP: hyperplastic polyp; ECO: expanded crypt opening; TBV: thick and branched vessels; NV: network vessels; WDC: white dendritic change.

## Data Availability

Patient data used to support the findings of this study are available from the corresponding author upon request. However, some of them are restricted by the institutional review board of Kyoto Prefectural University of Medicine.
